# Evaluating the Influence of Ferrule Preparation on Zirconia Endocrown Efficacy in Primary Molars: A 3D Finite Element Analysis

**DOI:** 10.1055/s-0045-1809534

**Published:** 2025-07-05

**Authors:** Ali Sayed Ali Bayiumy, Mohamed AboElkasme Wakwak, Salem Abdel Hakim Salem, Mohammed Said AbdAllah AbuSamadah, Mahmoud El-Said Ahmed Abd El-Aziz, Yasser R. Souror

**Affiliations:** 1 Department of Dental Biomaterials, Faculty of Dentistry, Assiut University, Assiut Governorate, Egypt; 2 Department of Pediatric Dentistry, Faculty of Dentistry, Al-azhar University, Assiut, Egypt; 3 Department of Pediatric Dentistry, Faculty of Dentistry Al-Azhar University, Cairo, Egypt; 4 Department of Dental Biomaterials, Faculty of Dental Medicine, Al-Azhar University, Assiut, Egypt; 5 Department of Pediatric Dentistry, Faculty of Dental Medicine, Misr University for Science and Technology, Cairo, Egypt

**Keywords:** endocrowns, primary molar, zirconia crown, ferrule height, finite element analysis

## Abstract

**Objective:**

This article assesses the effect of different ferrule preparations on the stress distribution in the primary second molar restored with zirconia crown.

**Materials and Methods:**

Four finite element models were created to simulate different ferrule heights: M1 (0 mm), M2 (1 mm), M3 (1.5 mm), and M4 (2 mm). A lower primary second molar was scanned to create a solid model, which was imported into finite element analysis software. Simulations included varying ferrule heights, material properties, and meshing. Models were subjected to 330 N occlusal loads at vertical, oblique, and lateral angles.

**Results:**

The analysis revealed that stress within the endocrown body increased with greater ferrule height under vertical loading. Conversely, stress levels decreased with increased ferrule height under oblique and lateral loads. Under vertical load, peak stresses were recorded as follows: endocrown body (219.5 MPa for M1), cement layer (11.7 MPa for M1 and M4), remaining tooth (36 MPa for M1), cortical bone (59.7 MPa for M1), and cancellous bone (8.7 MPa for M1 and M4). Under oblique load, stress values increased as follows: cement layer (62.9 MPa for M4), remaining tooth (59 MPa for M1), and endocrown body (203 MPa for M1). Under lateral load, stress values increased as follows: endocrown body (321 MPa for M1), cement layer (100 MPa for M4), remaining tooth (94 MPa for M1), cortical bone (154 MPa for M1), and cancellous bone (15 MPa for M1).

**Conclusion:**

Ferrule height significantly influences stress distribution in the tooth structure and supporting bone. Higher ferrule heights enhance structural stability by reducing stress on underlying components.

## Introduction

Managing extensively damaged primary molars remains a significant challenge in pediatric dentistry. Endodontically treated primary molars require restorative solutions that are not only durable and reliable but also capable of maintaining function and aesthetics until the natural exfoliation of the tooth. Traditionally, full-coverage restorations have been the preferred approach, with options including stainless steel crowns (SSCs), preveneered SSCs, and all-ceramic crowns like zirconia crowns. Each of these restorative solutions comes with its own set of advantages and limitations. 1

SSCs are known for their durability and ease of placement, making them a popular choice. However, they often fall short in terms of aesthetics, which can be a concern for parents and patients. 2

Preveneered SSCs improve the aesthetic outcome by adding a tooth-colored veneer. However, preveneered SSCs often face challenges, including veneer chipping or detachment, as well as difficulty achieving optimal marginal adaptation. A poor marginal fit can lead to a compromised tooth–restoration interface and increased risks of secondary caries. 3

All-ceramic crowns, such as zirconia crowns, offer superior aesthetics and positive gingival tissue response with lower plaque accumulation, yet require meticulous and extensive tooth preparation to achieve an accurate fit, a procedure that can be challenging in primary molars due to their smaller size and complex anatomical contours. 4 Additionally, the higher cost of zirconia crowns poses a consideration for both dental practices and patients, as these crowns are often more expensive than SSCs and preveneered alternatives. This factor may limit their accessibility and usage, particularly in resource-limited settings. 5

Endocrowns are single-piece, monolithic restorations crafted from full-composite or full-ceramic materials, designed to restore the coronal structure of an endodontically treated tooth. They achieve macromechanical retention by anchoring into the pulp chamber and cavity margins, while micromechanical retention is provided through adhesive cementation. 6 These design features allow endocrowns to provide both strength and stability within the remaining tooth structure, making them effective for conserving tooth structure in restorative procedures. 7 Additionally, endocrowns provide a cost-effective and aesthetic alternative, particularly for patients with a lower willingness-to-pay threshold. 8

Endocrowns are created through heat pressing or the more recent and widely adopted computer-aided design/computer-aided manufacturing (CAD/CAM) technology. The heat pressing method involves casting from a traditional impression, using wax patterns and ceramic ingots for a reliable fit. CAD/CAM, however, uses digital impressions and CAD and milling, allowing for faster, single-session fabrication with enhanced accuracy and data storage benefits. This method has become popular due to its efficiency and precision in producing durable restorations. 9 Endocrowns, while advantageous for treating structurally compromised teeth, rely heavily on the choice of materials to achieve optimal durability and aesthetics. The selected crown material directly impacts the restoration's resistance to wear, fracture, and color stability, with popular choices including ceramics and composite resins. Ceramics, for instance, offer superior hardness and natural aesthetics but may be more prone to brittleness. Composite resins, on the other hand, provide better flexibility, making them more forgiving under stress but potentially less durable over time. 10 The design and extent of tooth preparation are critical for retention and stress distribution, while the magnitude and direction of occlusal forces significantly affect the stress experienced by both the endocrown and the underlying tooth. 11 Furthermore, the ferrule, a circumferential band of crown material surrounding the cervical portion of the tooth, has been shown to enhance fracture resistance and prevent dislodgement by providing additional support at the tooth–restoration interface. 12

Finite element analysis (FEA) is an advanced computational tool used to simulate the behavior of materials and structures under various physical conditions. This method involves advanced mathematical calculations to create computer-generated models, enabling precise predictions of how an object will perform across different scenarios. 13

In dentistry, FEA has been extensively applied to study the biomechanical behavior of teeth and restorative materials, including how these structures respond to forces from activities like chewing, grinding, and biting. This approach provides detailed visualization and quantification of stress distribution within dental structures, allowing researchers to identify potential failure points and optimize restorative designs without relying on physical prototypes. 14 By modeling complex interactions between tooth tissues and restorative materials, FEA offers critical insights that can enhance the durability and reliability that predict the longevity of dental restorations. 15

Applying FEA to the study of ferrule design in endocrowns contributes to a better understanding of stress distribution and failure prevention. This insight aids in developing restorations that withstand occlusal forces more effectively, ultimately improving clinical outcomes. 16 Several studies have explored the effect of ferrule height on the efficacy of different crown materials, including metal, ceramic, and composite crowns. 17 18 Much of the previous research has focused on metal and ceramic crowns, while the use of zirconia crowns has gained prominence due to their aesthetic and mechanical properties. However, limited research has been conducted on the specific effects of ferrule height on zirconia crown restorations, particularly in primary molars.

In scenarios involving severely damaged primary teeth—such as primary molars that have undergone pulpotomy or pulpectomy due to extensive caries—endocrowns offer a conservative and efficient restorative solution. When minimal tooth structure remains, traditional post-and-core buildup and crown techniques may lack retention and are unsuitable due to the short roots and physiological root resorption of primary teeth. Endocrowns utilize the pulp chamber for retention, eliminating the need for a post and core, reducing adhesive interfaces, and preserving more tooth structure. They also minimize preparation and chair time, offering significant advantages in pediatric care. 19

This study aims to investigate how different ferrule heights influence stress distribution within the tooth–restoration complex and identify potential failure points under simulated occlusal loading conditions.

Null hypothesis (H _0_ ): There is no significant effect of ferrule height on the stress distribution in primary second molars restored with zirconia crowns.

## Materials and Methods

**Fig. 1:**
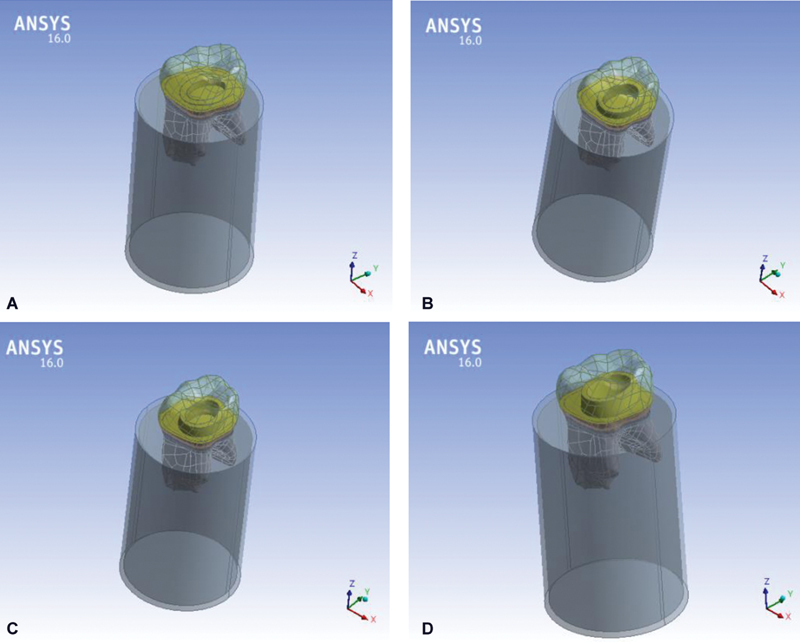
The four models: ( **A** ) Model #1: 0 mm ferrule, ( **B** ) Model #2: 1 mm ferrule, ( **C** ) Model #3: 1.5 mm ferrule, and ( **D** ) Model #4: 2 mm ferrule.

**Fig. 2:**
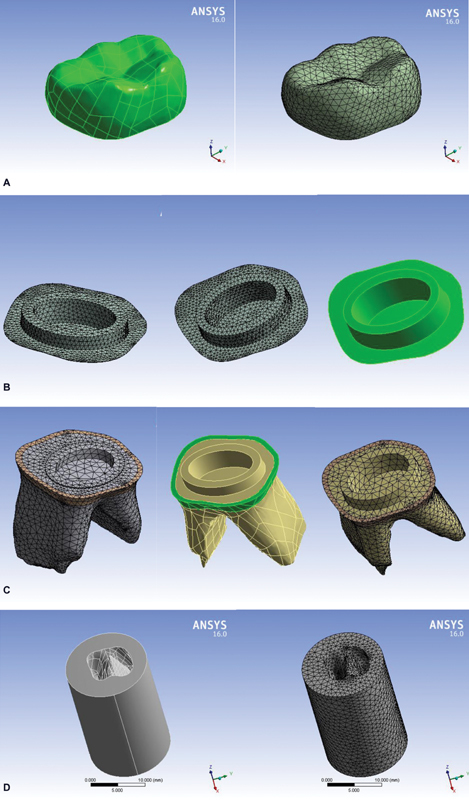
Screen shots for models' components and its mesh: ( **A** ) crowns, ( **B** ) cement layers, ( **C** ) tooth structure, ( **D** ) cancellous bone, ( **E** ) cortical bone, and ( **F** ) Loading points.

In this study, four finite element models of a primary mandibular second molar were developed. The modelling process began with laser scanning of a recently extracted, intact tooth. The tooth was removed due to a periodontal condition, and the extraction was performed following parental consent. 20 The outer geometry of the tooth was captured using a laser scanner (Geomagic Capture, 3D Systems, Cary, North Carolina, United States), which produced a data file containing a point cloud in STL format. This stereolithography format represents the surfaces of a solid model using a network of triangles. Intermediate processing was performed using Rhino 3.0 software (McNeel Inc., Seattle, Washington, United States) to trim the generated surfaces, creating a solid, closed geometry of the tooth. The final model was then exported to a FEA program in STEP file format. 21 Simultaneously, cortical and cancellous bone models were constructed utilizing commercial CAD software (AutoDesk Inventor, version 8.0, Autodesk Inc., San Rafael, California, United States). The simplified bone geometry consisted of two coaxial cylinders: the inner cylinder, representing cancellous bone, had a diameter of 14 mm and a height of 22 mm, occupying the inner space of the outer cylinder. The outer cylinder, representing cortical bone, was modeled as a 1-mm-thick shell with an external diameter of 16 mm and a height of 24 mm. 22 The endocrown was designed with a 2-mm thickness, and the access cavity had an elliptical shape with dimensions of 6 mm width, 4 mm height, and 2 mm depth, with walls tapered at a 5-degree angle. A cement layer, 20 to 40 μm thick, was placed beneath the endocrown. 14 Boolean operations in the ANSYS environment version 16.0 (ANSYS Inc., Canonsburg, Pennsylvania, United States) were used to complete the geometry (see Fig. 1 ). Full bond was assumed between all geometries, and all materials in each model were treated as isotropic, linear, and elastic, as specified in Table 1 . Model components were meshed using the three-dimensional brick solid element “SOLID187,” which provides three degrees of freedom (translation along the main axes). A meshing convergence test (with an error margin of less than 3%) was conducted by applying a test load across different mesh densities to ensure the accuracy of the discrete model results. Node and element count for the models are provided in Table 2 , with examples of the meshed components shown in screenshots from ANSYS (see Fig. 2 ).

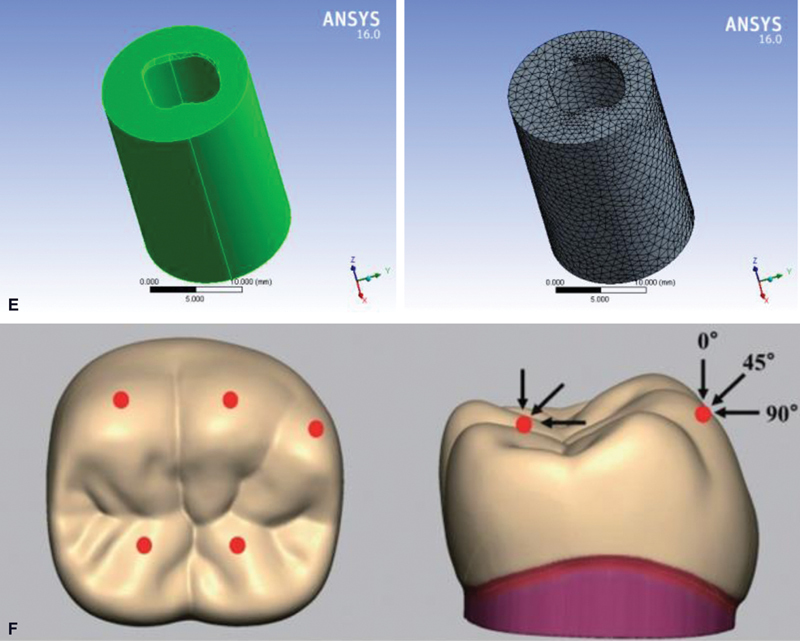



**Table 1:** Material properties of used in the finite element model(s)

Material	Young's modules [MPa]	Poisson's ratio
Zirconia crown	210,000	0.30
Cement: resin cement	7,000	0.27
Dentine	18,600	0.31
Enamel	84,100	0.30
Cortical bone	13,700	0.30
Cancellous bone	1,370	0.30

**Table 2:** Mesh density of the four models' components

	Model 1:	Ferrule 0 mm	Model 2:	Ferrule 1 mm	Model 3:	Ferrule 1.5 mm	Model 4:	Ferrule 2 mm
Material	Number of elements	Number of nodes	Number of elements	Number of nodes	Number of elements	Number of nodes	Number of elements	Number of nodes
Crown	17,745	26,330	12,113	18,563	11,607	17,863	11,749	18,180
Cement layer	11,354	23,247	8,705	17,949	9,270	19,083	10,056	20,673
Dentine	60,160	85,975	60,684	86,374	60,563	86,121	56,909	81,292
Enamel	3,070	5,414	3,070	5,414	3,070	5,414	3,070	5,414
Cortical bone	10,946	21,481	10,946	21,481	10,946	21,481	10,946	21,481
Cancellous bone	167,329	230,131	167,329	230,131	167,329	230,131	167,329	230,131

Three loading scenarios were applied to each model, involving a total load of 330 N in three directions: vertically, obliquely at 45 degrees, and laterally. Loading was applied equally to three points on the outer inclines of the buccal cusps and two points on the inner inclines of the lingual cusps ( Fig. 2F ). The models were verified against similar studies, 14 19 before analysis results were extracted.

The lowest plane of each model was fixed as a boundary condition. Twelve cases of linear static analyses were performed on a workstation (HP Z820, Dual Intel Xeon E5–2660, 2.2 GHz processors, 64GB RAM).

The analysis results are presented as graphical distributions of deformations, strains, and stresses. These distributions highlight critical locations that may experience high stress, significant deformation, or potential failure, indicated by red arrows. Conversely, minimum values, which may also represent critical conditions, are marked with blue arrows to highlight their locations.

Conclusions can be withdrawn by comparisons between the four models' components under the same loading condition.

## Results

**Fig. 3:**
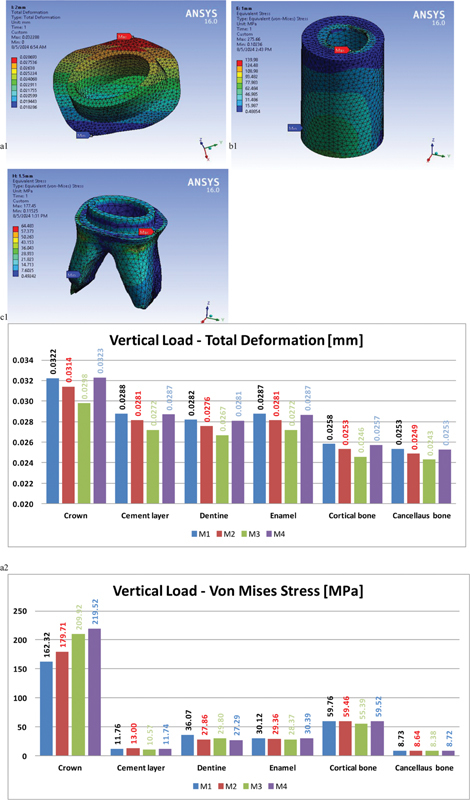
Sample results and comparisons; (a1, a2, a3) model #4 cement layer total deformation distribution, total deformation, and von Mises stress values comparison among the three models, (b1, b2, b3) model #3 tooth structure von Mises stress distribution, total deformation, and von Mises stress values comparison among the three models, (c1, c2, c3) model #2 cortical bone von Mises stress distribution, total deformation, and von Mises stress values comparison among the four models.

Under vertical loading ( Fig. 3 a1 , a2 , and a3 ), endocrown body stresses increased by increasing ferrule height. While cement layer showed varying reaction, that minimum value of cement von Mises stress was found with 1.5 mm ferrule height model by approximately 10.6 MPa. The maximum stress values appeared on 1 mm ferrule height model “13 MPa,” while different cement layers under vertical load showed less variations.

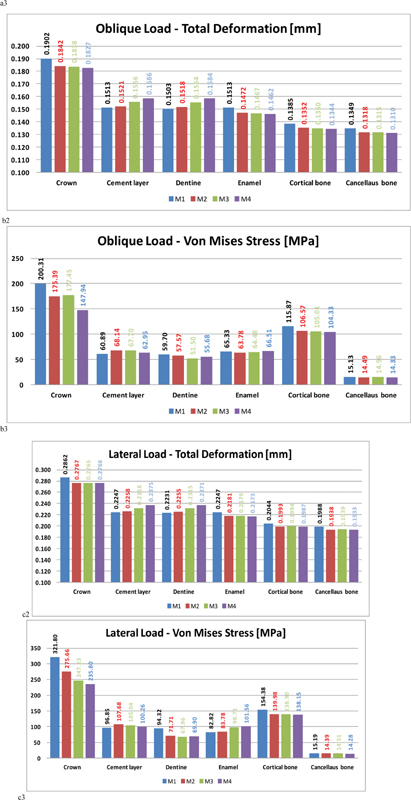



Remaining tooth “dentine” received the highest stresses by approximately 30 MPa with no ferrule while different ferrule heights did not show significant variation “less than 4%,” while 1.5 mm ferrule height showed the lowest stresses values. The 1.5-mm ferrule model showed the advantage of exerting lowest stress value, with cortical and cancellous bone showing approximately 7 and 5% less stresses in comparison to no ferrule model, which showed the highest values.

All deformation variation between different models did not exceed 5 µm for each component, and stresses were within acceptable values for the component materials, that no direct failure or to be expected by repeating this vertical load.

Oblique load results ( Fig. 3 b1 , b2 , and b3 ) showed considerable differences in comparison to vertical loading cases, that the deformation and stress values showed increase by about six and two times respectively. One exception was noticed on cement layer stresses, which showed about five times increase. For each component the differences in deformation among the four models did not exceed 10 µm.

Endocrown body showed decreasing trend with increasing ferrule height. Additionally, this trend extended to all other components but with lower differences.

One exception was recorded on cement layer, that no ferrule model exerted the lowest stresses on cement layer by approximately 3% less than the longest ferrule (showed ∼63 MPa), while the model with 1 mm ferrule showed approximately 68 MPa. Another point also recorded on remaining tooth, that model with 1.5 mm ferrule height showed slightly lower stresses, by approximately 3 to 7% then the 2-mm ferrule height model.

Lateral load results ( Fig. 3 c1 , c2 , and c3 ) showed increase in deformations and stresses by approximately 50% in comparison to oblique cases. Two exceptions were noted on bone stresses, that cancellous bone showed nearly no change while cortical bone von Mises stress increased by approximately 30% under lateral load in comparison to oblique one.

Similar to oblique loading, the endocrown body showed decrease in its von Mises stress by increasing ferrule height under lateral loading. No ferrule model showed the lowest von Mises stress, approximately 97 MPa, while the three models with ferrule heights 1, 1.5, and 2 mm showed approximately 108, 105, and 100 MPa, respectively.

Cement layer results followed the same trend as that found under oblique loads, while the stress values were higher by approximately 50%.

Remaining tooth under lateral loads showed the highest values of von Mises stresses in comparison to vertical and oblique loads.

Again, lateral and oblique loads showed similarity, that, cortical bone under no ferrule crown showed approximately 10% more von Mises stress in comparison to the 2-mm ferrule height model. While the other ferrule heights showed less than 3% von Mises stress in comparison to the 2-mm ferrule height model.

## Discussion

This study aimed to investigate the impact of ferrule height on the structural performance of endocrowns in primary molars using FEA. The results demonstrate that ferrule height significantly affects stress distribution across the tooth, crown, and supporting structures. Specifically, increased ferrule height improved resistance to lateral and oblique forces but also led to higher stresses under vertical loads.

The lower second primary molar was selected in this study as it is considered more vulnerable to caries development, plays a crucial role in maintaining the occlusal relationship, and is often subjected to significant occlusal forces during mastication. 23

Although a 330-N force is higher than the average maximum bite force typically observed in children, substantial variability exists due to factors such as age, jaw development, and specific functional tasks. This increased load can account for peak values found in certain cases or during specific activities, ensuring that restorations are tested under the potential upper thresholds for young patients. 24

In this study, the pulp chamber was leveled to create a standardized, flat floor, ensuring uniform seating of the endocrown restoration. The access cavity was designed with an elliptical shape, featuring dimensions of 6 mm in mesiodistal width, 4 mm in occluso-pulpal depth, and 2 mm in floor-to-ceiling height. This leveling strategy replicates typical clinical preparation, minimizes geometric variations, and ensures a consistent cement layer thickness across all specimens, thereby allowing accurate evaluation of ferrule height to impact mechanical performance. The standardization also promotes predictable stress distribution within both the restoration and the underlying tooth structure during mechanical loading. The pulp chamber extension angle was set at a 5-degree divergence, consistent with the taper of the access cavity walls, as this angle influences the mechanical behavior of the endocrown by affecting both retention and stress distribution. Our findings demonstrate that increasing ferrule height significantly enhances the structural support of endocrowns, particularly under oblique and lateral loading conditions, by improving their resistance to bending forces. This reinforcement effect can be attributed to the ferrule functioning as a circumferential stabilizing band, which optimizes stress distribution and enhances the biomechanical integrity of the restoration. By mitigating bending moments and promoting more uniform load dispersion across the tooth–restoration interface, a greater ferrule height contributes to improved longevity and fracture resistance of endocrowns. These results are consistent with the findings of AboElhassan et al. 25

Conversely, under vertical loading conditions, an increase in ferrule height was associated with elevated stress concentrations. This effect can be attributed to the reduction in the endocrown body volume above the ferrule, which limits the available material for absorbing and dissipating compressive forces. A decreased crown volume results in a diminished capacity to distribute vertical stresses effectively, potentially compromising the structural performance of the restoration. This observation is consistent with the findings of Kutesa-Mutebi and Osman, who reported that the ferrule effect does not significantly enhance fracture resistance under vertical compressive loading. 26 These results suggest a complex relationship between ferrule height and the mechanical performance of endocrowns, emphasizing the importance of careful ferrule design in clinical practice to optimize durability and function. 27

Regarding stress distribution within the cement layer, both the endocrown model without a ferrule and the model featuring a 2-mm ferrule exhibited the lowest stress levels. In contrast, models with 1.5 and 1 mm ferrules showed progressively higher stress concentrations. This increase in stress for thinner ferrule models is likely due to the altered cement geometry at the upper margin of the ferrule, where the less robust cement material is subjected to greater stress intensities. These results corroborate the findings of Tribst et al, who reported that a 2-mm ferrule is more effective than a 1-mm ferrule in minimizing stress within the cementing layer. 28

The remaining tooth structure, particularly the dentine, showed a marginal advantage in the 1.5-mm ferrule height model compared with the 2-mm model under lateral and oblique loading. Both the 1.5- and 2-mm ferrule height models outperformed the no-ferrule model, demonstrating enhanced stress resistance and a more favorable force distribution across the tooth. This finding aligns with the work on the structural benefits of moderate ferrule height. 12

Interestingly, the enamel ring of the remaining tooth showed increased stress with greater ferrule height under oblique and lateral loads, yet the opposite trend was observed under vertical loading. Vertical forces tend to open the ferrule walls, resisted by the enamel ring, while oblique and lateral forces decompose into vertical and lateral components, allowing the enamel to provide support to the ferrule walls. However, the lateral loads generated the highest stress in the remaining tooth compared with vertical and oblique loads; the stress levels remained within physiological limits. 29

Cortical bone stress levels were significantly influenced by load direction. Under lateral loading, the cortical bone experienced stresses nearing the upper limits of physiological tolerance, which could risk damage. However, the cortical bone tolerated oblique and vertical loads more effectively, with stress remaining below harmful thresholds. Increasing ferrule height slightly reduced cortical bone stress by distributing load over a larger area, as observed when the direction of loading shifted from lateral to oblique to vertical. 30

Lastly, cancellous bone experienced nearly double the von Mises stresses under oblique and lateral loading compared with vertical loading, yet maintained stress levels within physiological limits. This indicates that, although oblique and lateral forces impose significantly higher stresses on cancellous bone, it remains structurally sound and capable of enduring these forces without risk of failure. 31 32

The results demonstrate that ferrule height significantly affects stress distribution across the tooth, crown, and supporting structures. Specifically, increased ferrule height improved resistance to lateral and oblique forces but also led to higher stresses under vertical loads.

The findings have important clinical implications. Ferrule heights between 1.5 and 2 mm showed superior performance by optimizing stress management and enhancing structural stability. The 1.5-mm ferrule model minimized stress concentration within the cement layer, while taller ferrules provided better force distribution across the tooth–restoration complex. These outcomes suggest that clinicians should prioritize ferrule heights of 1.5 to 2 mm for pediatric zirconia crowns to balance mechanical durability and stress reduction.

The results also underscore the need for careful customization of ferrule height based on individual patient loading scenarios to minimize failure risks.

## Conclusion

In conclusion, ferrule height critically influences the mechanical behavior of endocrowns in primary molars. Customizing ferrule height based on clinical demands can enhance restoration longevity, with ferrule heights between 1.5 and 2 mm recommended for optimal performance. Future research should incorporate diverse force applications and clinical trials to strengthen the evidence base for best practices in pediatric crown restorations.

This research contributes to understanding how ferrule height affects the biomechanical behavior of endocrowns in pediatric patients. Although FEA offers powerful modelling capabilities, it is based on assumptions that may not fully represent biological complexities such as periodontal ligament representation and apply loading on specific points. The study lacks empirical validation against clinical outcomes, which could limit the applicability of these findings. Future research should focus on longitudinal studies and incorporate a broader range of loading conditions to better simulate the diverse forces encountered by children in daily function.
